# Sixteen kiwi (*Apteryx* spp) transcriptomes provide a wealth of genetic markers and insight into sex chromosome evolution in birds

**DOI:** 10.1186/s12864-016-2714-2

**Published:** 2016-05-26

**Authors:** Kristina M. Ramstad, Hilary C. Miller, Gabriel Kolle

**Affiliations:** Department of Biology and Geology, University of South Carolina Aiken, 471 University Parkway, Aiken, SC 29801 USA; Allan Wilson Centre, School of Biological Sciences, PO Box 600, Victoria University of Wellington, Wellington, 6140 New Zealand; Biomatters Ltd, Level 2, 18 Shortland Street, Auckland Central, Auckland 1010 New Zealand; Illumina Australia, Scoresby, VIC Australia

**Keywords:** Transcriptome, Kiwi, *Apteryx*, Sex chromosome evolution, *De novo* assembly

## Abstract

**Background:**

Kiwi represent the most basal extant avian lineage (paleognaths) and exhibit biological attributes that are unusual or extreme among living birds, such as large egg size, strong olfaction, nocturnality, flightlessness and long lifespan. Despite intense interest in their evolution and their threatened status, genomic resources for kiwi were virtually non-existent until the recent publication of a single genome. Here we present the most comprehensive kiwi transcriptomes to date, obtained via Illumina sequencing of whole blood and *de novo* assembly of mRNA sequences of eight individuals from each of the two rarest kiwi species, little spotted kiwi (LSK; *Apteryx owenii*) and rowi (*A. rowi*).

**Results:**

Sequences obtained were orthologous with a wide diversity of functional genes despite the sequencing of a single tissue type. Individual and composite assemblies contain more than 7900 unique protein coding transcripts in each of LSK and rowi that show strong homology with chicken (*Gallus gallus*), including those associated with growth, development, disease resistance, reproduction and behavior. The assemblies also contain 66,909 SNPs that distinguish between LSK and rowi, 12,384 SNPs among LSK (associated with 3088 genes), and 29,313 SNPs among rowi (associated with 4953 genes). We found 3084 transcripts differentially expressed between LSK and rowi and 150 transcripts differentially expressed between the sexes. Of the latter, 83 could be mapped to chicken chromosomes with 95% syntenic with chromosome Z.

**Conclusions:**

Our study has simultaneously sequenced multiple species, sexes, and individual kiwi at thousands of genes, and thus represents a significant leap forward in genomic resources available for kiwi. The expression pattern we observed among chromosome Z related genes in kiwi is similar to that observed in ostriches and emu, suggesting a common and ancestral pattern of sex chromosome homomorphy, recombination, and gene dosage among living paleognaths. The transcriptome assemblies described here will provide a rich resource for polymorphic marker development and studies of adaptation of these highly unusual and endangered birds.

**Electronic supplementary material:**

The online version of this article (doi:10.1186/s12864-016-2714-2) contains supplementary material, which is available to authorized users.

## Background

Species of conservation concern have arguably the greatest need for genomic tools but can present significant challenges to their development and use. Sampling large numbers of individuals or obtaining fresh tissue samples is often impossible when working with at-risk species due to their rarity and protected status [1, 2]. The species of interest may also be phylogenetically divergent and lack a closely related reference genome, making genomic annotation and assembly difficult [3]. In addition, many at-risk populations will have experienced genetic bottleneck effects, small population size, and inbreeding, which can result in low levels of genetic diversity and strong gametic disequilibrium and make discovery of large numbers of independent polymorphic markers challenging. These difficulties initially slowed the development and use of genomic tools for endangered species, and the full application and power of conservation genomics to improve species management is only now becoming evident [4–9]. At the same time, the field of genomics has placed emphasis on broad evolutionary comparisons rather than on delineating genomic variation within species. Genomic studies often deeply sequence one to three individuals of a single species for use in comparisons among orders or families. Conservation management, however, occurs at the population level, with a focus on understanding and maintaining genetic diversity within and between species.

Transcriptome sequencing allows elucidation of coding gene sequences without the requirement of deep genome sequencing. Next-generation sequencing techniques allow for the collection of mRNA sequences from small amounts of tissue in parallel across individuals and species [2], at a depth and coverage that allow *de novo* transcriptome assembly even in the absence of a closely related genome [10]. Tissue samples can be collected non-lethally from a single tissue type, such as skin, and still contain sequences from large numbers of genes with diverse functions [2]. Alternatively, samples from diverse tissues can be taken from individuals that have recently died of natural causes, and still provide enough high quality sequence data to build a transcriptome [1]. This provides exciting new possibilities for transcriptome assembly and characterization from rare and endangered species, and thus unprecedented possibilities for marker discovery, gene characterization, and comparative genomics of these species as well.

Kiwi (Family Apterygidae) are ratite birds endemic to New Zealand. They are of great evolutionary interest because they represent the most basal extant avian lineage (paleongaths) and possess numerous biological attributes that are unusual or extreme among birds. These include large egg size, paired functional ovaries, low metabolic rate, strong olfaction, and lack of color vision [11, 12] as well as flightlessness (present in < 1% of extant birds) [13], long lifespan (potentially more than 80 years) [14–16], and nocturnality (less than 3% of avian species) [11, 17]. Kiwi are also of great conservation concern, with four of their five species listed as threatened by the International Union for Conservation of Nature [18]. Kiwi comprise two clades, the brown and spotted kiwi, which diverged from one another approximately 16 million years ago [19]. The rowi (*Apteryx rowi*) is a brown kiwi and the rarest kiwi species. They comprise a single population of approximately 430 birds that rebounded from a population size of approximately 150 birds in the mid 1990’s [20]. A third of adult rowi do not breed and annual hatching success in the wild is 16% at best [21]. The little spotted kiwi (LSK, *A. owenii*) is the second rarest kiwi species. All extant LSK arose from at most five birds placed on Kapiti Island approximately 100 years ago. Despite rapid population growth, LSK are depauperate in genetic diversity at important disease resistance genes [22], show signs of inbreeding depression [23], and continue to experience genetic erosion [24]. Annual hatching success for wild LSK can be as low as 27% [23]. Reduced fertility and hatching are often the first signs of inbreeding depression in small, isolated avian populations [25–29]. Thus, poor reproductive success may have an underlying genetic basis in LSK and rowi, potentially exacerbated by the strong genetic drift they experienced during recent genetic bottlenecks [24] and subsequent inbreeding due to small population size [23].

Intense conservation measures have been taken with kiwi, including captive incubation, translocation, and predator control programs [20]. Genomics research could greatly enhance these efforts by providing robust pedigrees, assessing levels of hybridization, investigating possible genotype-phenotype correlations and estimating relatedness and inbreeding coefficients. However, few genomic resources have been available for kiwi until recently [11, 30] and none have sequenced more than three birds representing a single species and sex. Indeed, all genomic studies of kiwi to date have focused on the relatively common (~25,000 individuals) and widespread North Island brown kiwi (NIB, *A. mantelli*) [20] without regard to the genetic divergence expected between clades or species.

In this study, we used Illumina sequencing to assemble transcriptomes of 16 kiwi solely from samples of whole blood. Our primary objective was to define suites of functional and neutral genetic markers for brown and spotted kiwi in a single sequencing effort and without having to sacrifice any birds. Specifically, we sought to identify (1) markers in genes of known function that display coding DNA sequence variation between species and among individuals, (2) putatively neutral genetic markers that display DNA sequence variation between species and among individuals and (3) markers in genes that show differential expression between species and sexes. For each species, we assembled 200 million paired end reads into approximately 200 thousand contigs, of which approximately 20 thousand contained predicted coding gene sequences with mean open reading frame (ORF) lengths greater than 900bp. We were able to annotate more than half of these ORF containing contigs by comparison with sequences in the NCBI non-redundant protein database. In addition, we have catalogued an extensive list of sequence variation, found many genes that are differentially expressed between species and sexes, and identified polymorphic markers within genes related to reproduction, growth, development, behavior, and disease resistance. Our results will help delineate the structure, function and expression of kiwi genes, and provide a powerful resource for further studies of kiwi ecology, management, and evolution.

## Results and discussion

### De novo assembly and annotation

We sequenced mRNA libraries from the blood of eight LSK and eight rowi using an Illumina HiSeq and obtained more than 348 and 502 million paired reads from LSK and rowi, respectively. Raw HiSeq reads are available at the NCBI Short Read Archive under accession number SRP074490. While a more comprehensive transcriptome could have been obtained from sequencing mRNA from various tissues, our non-lethal sampling means that we can continue to monitor our study birds to explore additional genotype-phenotype relationships in the future. A possible limitation of sequencing only blood is the high level of hemoglobin transcripts often encountered (50–70%) [31, 32]. However, we found that only 18% of read pairs (*n* = 150,157,518) in the final data (*n* = 851,254,015) mapped to hemoglobin (Table 1), enabling us to still assemble comprehensive transcriptomes from a moderate number (>700 million in total) of reads.

**Table 1:** Summary of transcript assembly for LSK and rowi

	LSK	Rowi
Total number of read pairs	348,651,735	502,602,280
Number of reads mapped to hemoglobin	68,514,883	81,642,635
Total number of contigs	215,094	207,470
Number of contigs with predicted ORF > 100aa	20,152	23,830
Mean transcript length (ORF > 100aa)	2,225bp	2,847bp
Mean ORF length (ORF > 100aa)	917bp	1,129bp
Number of possible chicken orthologues	7,908	8,631

Greater than 20 thousand contigs in each of LSK and rowi have a full or partial open reading frame (ORF) greater than 100 amino acids and thus a predicted protein coding sequence. Within species, approximately 8000 of these contigs show strong homology to chicken protein sequences

After removal of the transcripts mapping to hemoglobin, two hundred million reads from each species were randomly selected for Trinity assembly from those remaining, and formed 215,094 and 207,470 contigs for LSK and rowi, respectively (Table 1). Of these, 20,152 LSK and 23,830 rowi transcripts had a predicted full or partial Open Reading Frame (ORF) greater than 100 amino acids, which we focused our analyses on subsequently. Using BLASTP, we found 7908 transcripts in LSK and 8631 transcripts in rowi with strong homology (>80%) to the chicken Ensemble protein database (Table 1).

BLASTN comparison determined that more than 16,000 contigs containing ORFs were shared between the two species with 99% average homology. Thus, the LSK composite assembly was used as the master reference in all subsequent analyses (Additional file 1). We found strong alignment between our composite LSK transcriptome and the recently published North Island brown kiwi genome [11]. Of our 20,152 transcripts, 97% (19,533) mapped to at least one contig from the genome assembly with 76% (15,315) mapping to greater than 90% of the entire contig length with > 99% identity.

**Fig. 1:**
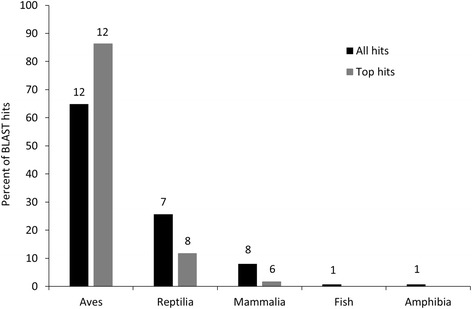
Percent of BLAST hits of kiwi mRNA transcripts against NCBI non-redundant (NR) database by vertebrate class. Numbers above bars indicate number of species represented within the percentage. Amphibians were represented solely by African clawed frogs (*Xenopus laevis*) and fishes by the African coelacanth (*Latimeria chalumnae*)

**Fig. 2:**
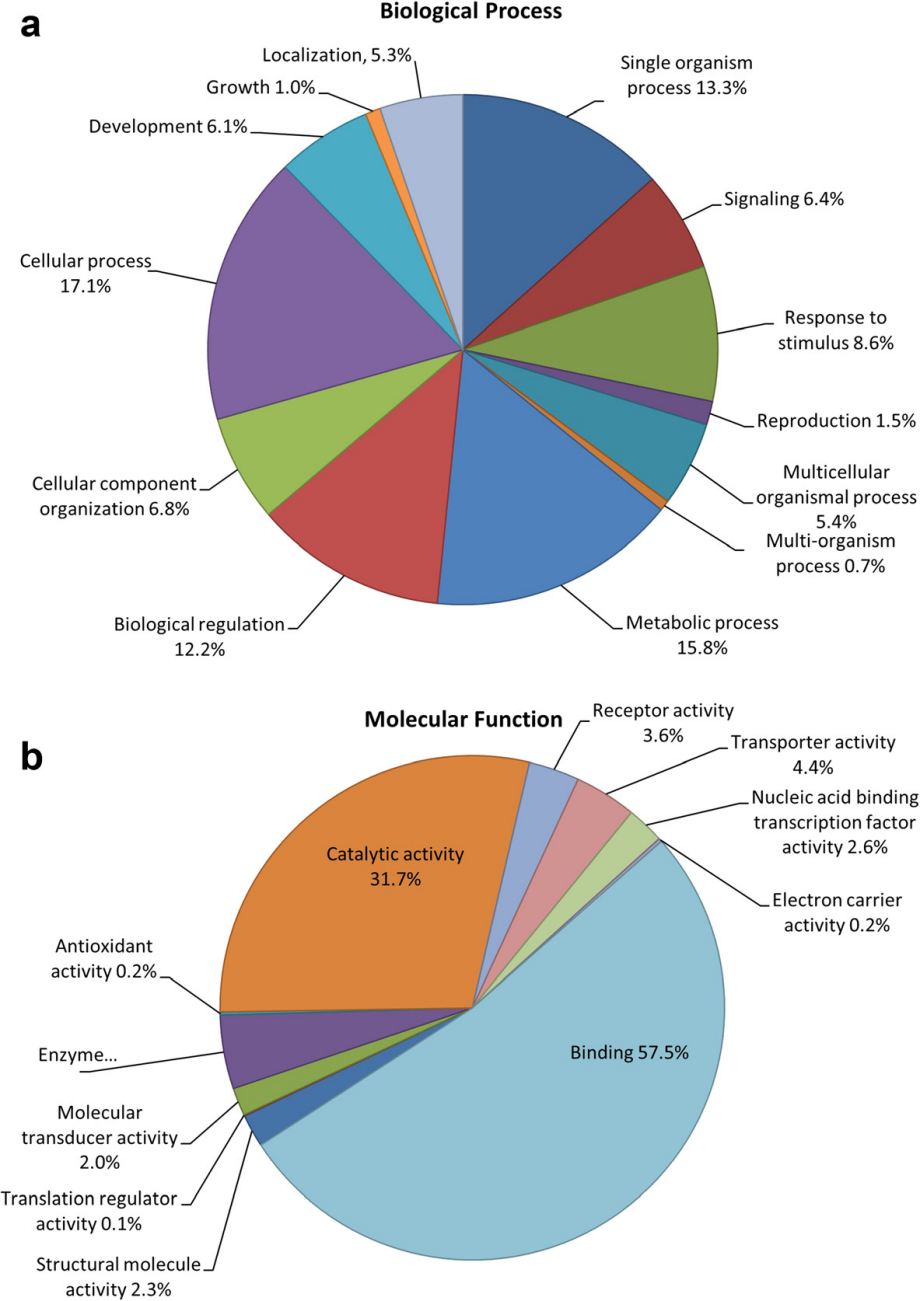
Level 2 gene ontology assignments of (**a**) biological process and (**b**) molecular function for little spotted kiwi transcripts

Sequences were further annotated by searching the NCBI non-redundant (NR) protein database using BLASTx (Additional file 2). Of the 20,152 unique LSK contigs containing an annotated ORF, 12,571 (62%) had matches with an e-value of less than 1e^−4^. Most of these matches were to birds and reptiles (Fig. 1), but a large diversity of species contributed to functional annotation. The highest number of top hits (alignments with the lowest e-value per sequence) were attributed to the mallard (*Anas platyrhynchos*, 1976 transcripts or 16%), chicken (1593 or 13%), peregrine falcon (*Falco peregrinus*, 1446 or 12%) and pigeon (*Columbia livia*, 1422 or 11%). Secondly, we used Blast2GO to assign Gene Ontogeny (GO) identifiers to 10,688 of the 12,571 sequences for which we obtained BLASTx results, or 53% of the 20,152 contigs originally blasted. More than half of the annotated genes were associated with biological regulation or cellular, metabolic, or single-organism processes (Fig. 2a). Greater than 80% of sequences annotated with a molecular function were associated with binding or catalytic activity (Fig. 2b), which is typical of transcriptomic studies regardless of taxa or tissue type sequenced [1–3, 30]. Importantly, we identified numerous sequences associated with growth (*n* = 355), reproduction (*n* = 554) and development (*n* = 2239).

### Sequence variation between and within species

One of the primary aims of this work was to define a highly confident sequence variant list that could be used for both population monitoring and genotype-phenotype associations in kiwi. To that end, we identified the full cohort of SNP variation across all samples by comparison to the LSK reference assembly. We found 83,222 and 434,504 SNPs present in at least one LSK or rowi, respectively. To find markers with high confidence, we filtered for SNPs present in at least 50 reads per individual. This resulted in a total of 27,170 SNPs in LSK and 120,035 SNPs in rowi. Of the SNPs found in rowi, 66,909 in 6164 contigs were exclusive to rowi and differentiate between the two species (Table 2; Additional file 3). A total of 12,384 (in 3089 contigs) and 29,313 (in 4953 contigs) SNPs in LSK and rowi met our criteria for a marker that could distinguish individuals within each species: (a) placement within contigs where SNP density was less than 1 per 200bp and (b) at least one individual with a SNP frequency lower than 5% and one with a frequency greater than 35% (Table 2; Additional files 4 and 5).

**Table 2:** Summary of SNPs discovered in LSK and rowi and those predicted to change protein coding sequences

	Total number	Number predicted to change amino acid
All SNPs found in LSK	27,170	5,434
All SNPs found in rowi	120,035	17,057
SNPs that differentiate LSK and rowi	66,909	7,418
SNPs that differentiate among LSK	12,384	1,832
SNPs that differentiate among rowi	29,313	4,466

SNPs that are unique to rowi and differentiate between species have greater than 50% coverage in each bird sequenced, a frequency of greater than 80% among all rowi sequenced, and less than 5% among sequenced LSK. SNPs that differentiate among LSK and rowi are within contigs with a SNP density less than 1 per 200bp and have a frequency of greater than 35% in at least one individual and less than 5% in another

We further categorized SNPs based on their likely effect on the gene coding sequence, finding 5434 in LSK and 17,057 in rowi that result in changes to amino acid coding sequences (Table 2). These potential markers map to genes present in each of the chicken chromosomes (Additional files 3, 4 and 5) and are candidates for targets of selection. In contrast, many of the SNPs we discovered are likely selectively neutral because they are positioned in untranslated regions of contigs or cause synonymous substitutions in coding regions.

Polymorphism in rowi was more than double that observed among LSK. This pattern is not likely due to differences in sequencing depth as there is little correlation between the number of species specific SNPs and the total number of reads sequenced for any individual kiwi (Pearson correlation coefficient = 0.09). More likely, the much greater SNP diversity detected in rowi, a pattern that has also been observed in mtDNA [33] and among microsatellite loci [34], reflects the differences in demographic history of the two species. LSK were reduced to five birds approximately 100 years ago and show numerous intense signs of this recent genetic bottleneck effect [24]. In contrast, rowi represent a very old and natural remnant population [33, 35] and though recently bottlenecked, retain greater ancestral genetic variation than LSK (K Ramstad, unpublished data) [34].

### Differential gene expression between species and sexes

**Fig. 3:**
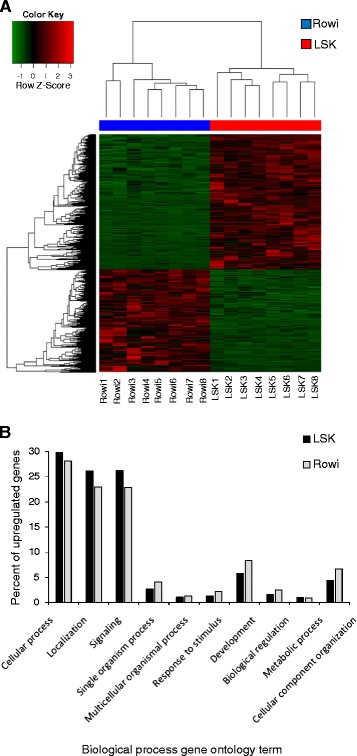
**(a) ** Heat map and cluster dendogram showing differential gene expression between little spotted kiwi (LSK) and rowi. Green and red indicate low and high expression, respectively; data are normalized per gene and sample. Mean upregulation of genes in LSK relative to rowi (1753 genes) and vice versa (1331 genes) was ~8 and 5 fold and ranged from 2 to 701 fold per gene. **(b) ** Biological process gene ontology (GO) assignments for genes significantly upregulated in LSK and rowi. Data are presented for GO terms representing 3% or greater of assignments for at least one species and are weighted by the number of genes with GO terms per species

By mapping reads to the reference and counting the number of reads mapping to each transcript, we found significant differences in patterns of gene expression between kiwi species and sexes (Additional file 6). A total of 3084 transcripts were differentially expressed by at least 2 fold between LSK and rowi (*β*-statistic > 0), including 1753 upregulated in LSK and 1331 upregulated in rowi (Fig. 3a; Additional file 7). Mean upregulation of genes in LSK and rowi was 8.1 fold (range 2.0 to 701.0) and 5.0 fold (2.0 to 242.5), respectively. Biological process GO terms were assigned to 524 genes upregulated in LSK and 643 genes upregulated in rowi. The distribution of genes across GO terms (weighted by the number of genes annotated) was similar in the two species, with genes involved in cellular processes, localization, and signaling more prevalent among genes upregulated in LSK and genes involved in development and cellular component organization more prevalent among genes upregulated in rowi (Fig. 3b).

**Fig. 4:**
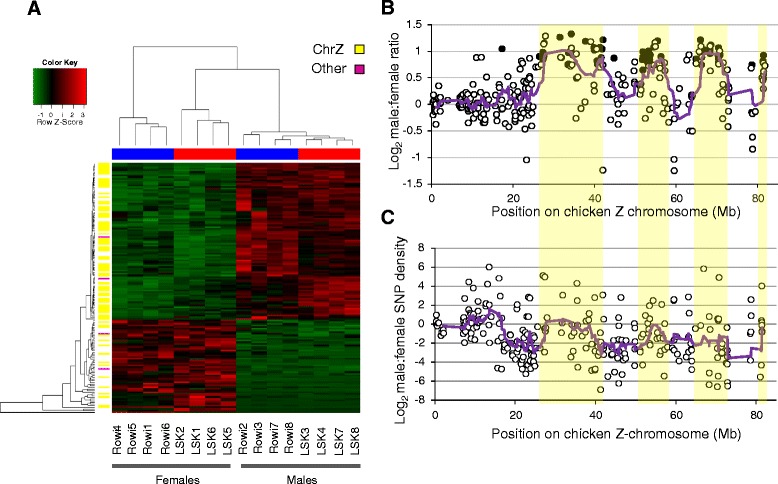
**a** Heat map and cluster dendogram showing differential gene expression between male and female kiwi. Green and red indicate low and high expression, respectively; data are normalized per gene and sample. Mean male-biased (94 genes) and female-biased (56 genes) expression was 2 and 11 fold and ranged from 1 to 101. Sixty-four of 66 genes showing male-biased expression mapped to chicken chrZ (**b**) Distribution of male to female expression ratio (**b**) and SNP density (**c**) across the mapping position of chicken chrZ, including genes with significant male-biased expression (black circles) and genes equally expressed between sexes (open circles). Lines represent a moving average across ten genes; yellow shading indicates chromosomal regions with both male-biased expression and SNP density and so likely outside the PAR

We did not use a 2 fold threshold in comparing expression differences between sexes as a 2 fold male-bias is expected of genes present on the Z chromosome (chrZ) but outside the pseudoautosomal region (PAR). We identified 150 differentially expressed genes with a *β*-statistic > 0 between sexes (88 of these with an expression difference ≥2 fold). Of these genes, 94 showed male-biased (38 genes >2 fold difference) and 56 (50 genes >2fold difference) showed female biased expression (Fig. 4a; Additional file 8). Male-biased expression ranged from 1.4 to 3.8 fold with a mean of 2.0 (range 2.0 to 3.8, mean 2.4 where expression > 2 fold), while female-biased expression ranged from 1.6 to 101.4 fold with a mean of 11.1 (range 2.0 to 101.4, mean 12.2 where expression > 2 fold). Of 150 genes differentially expressed between males and females, 83 (66 male-biased, 17 female-biased) could be mapped to a chicken chromosome; approximately 95% of these (n = 79) were syntenic to chrZ and none were syntenic to W (Fig. 4a; Additional file 8). The pattern of upregulation was particularly striking in males, with 64 genes syntenic to chicken chrZ and only two syntenic to other chromosomes. We were able to assign biological process GO terms to 52 genes showing male-biased expression and 21 genes exhibiting female-biased expression. The distribution of genes across GO terms was similar between the sexes and to that observed between species.

Expression differences observed between species may reflect differences in the age of birds or their environment. All of our samples came from birds greater than one year of age and well past the timeframe of gonadal differentiation marking the onset of sex specific expression in other ratites (42 days) [35]. However, our LSK samples were largely from adult birds inhabiting three locations, our rowi samples were exclusively from juveniles (1 to 3 years old) inhabiting a fourth location, and gene expression can differ markedly with age and between environments, even after gonad differentiation [36, 37]. Thus, the transcriptional variance observed between LSK and rowi could reflect physiological differences between adults and juveniles, environmental differences between the islands they occupy, or evolutionary divergence between the brown (including tokoeka (*A. australis),* North Island brown kiwi, and rowi) and spotted (including great spotted kiwi (A. *haastii*) and LSK) clades of kiwi [38]. In contrast, age and environmental effects cannot explain the observed sex-biased expression in LSK and rowi, because the sex ratio was equal within each age (three male and four female adults, five male and four female juveniles) and location (four male and four female rowi from Motuara Island, three male and three female LSK from Kapiti Island, one male and one female LSK from Long Island and one female LSK from Zealandia) sampled. Thus, differences in gene expression between the sexes likely reflect evolutionary differences between male and female kiwi.

Chickens and ratites exhibit strong chromosomal homology [35, 39–41] despite having diverged from a common ancestor 100 or more million years ago [30, 42, 43]. Indeed, highly conserved synteny among avian taxa allows chromosomal locations of kiwi genes to be predicted from that of chicken [35, 39]. In contrast, there is a huge diversity among birds in the degree of homomorphy and recombination between their Z and W chromosomes. The portions of the chromosomes that recombine define the PAR, where the same genes are present on both sex chromosomes and their sequences nearly identical [44]. Many avian lineages, including chicken, have highly differentiated sex chromosomes and a very small PAR, while more than two-thirds of the emu (*Dromaius novaehollandiae*) and ostrich (*Struthio camelus*) Z chromosomes recombine with the W defining a very large PAR [42, 44, 45]. This pattern is not common to all ratites however, as the size of the PAR varies among tinamou species (*Tinamus* spp) and can represent as little as 1% of chrZ (white-throated tinamou, *Tinamus guttatus*) [44, 46]. The extent of homomorphy in kiwi sex chromosomes is unknown, and there is considerable scope for kiwi to differ from other ratites in sex chromosome arrangement since they diverged from their nearest extant ratite relatives, the emu and cassowary (*Casuarius casuarius*), approximately 58mya [47].

Given that dosage compensation is essentially lacking in birds and females are the heterogametic sex [42, 44, 48], genes on chrZ are expected to be expressed in males at equal (within the PAR) to twice (outside the PAR) the level observed in females. Similarly, SNP density is expected to be equal between the sexes at chrZ genes within the PAR, but male-biased in genes outside the PAR for which females are always hemizygous [35]. In line with these expectations, we found clear bimodal expression of genes mapped to chicken chrZ (n = 300), with a minority displaying two-fold male-biased expression (log_2_ ~ 1) and the majority displaying equal expression in males and females (log_2_ ~ 0; Fig. 4b). Regions of chrZ with male-biased expression also displayed male biased SNP density, which is consistent with these regions being outside of the PAR (Fig. 4c).

Upregulated kiwi genes formed disjunct clusters across the chicken chromosome. This clustering has been observed previously in ostriches and attributed to extensive PARs exhibiting strong recombination and homomorphy [35, 49] in conjunction with chromosomal inversions since the evolutionary split of paleognaths and neognaths [50]. Our data are consistent with these observations, and suggest that kiwi exhibit an ancestral state of sex chromosome differentiation similar to that of the ostrich and emu, with large PARs and little differentiation between the Z and W chromosomes [35, 44, 49]. The much greater mean expression among female-biased genes may be due to sexual antagonism [35]. Mutations on ratite sex chromosomes are more likely to fall within than outside the PAR. If these mutations are also sexually antagonistic, they cannot be restricted to the sex they benefit via restricted recombination between the sex chromosomes. Strongly sex-biased expression, however, can moderate the influence of these genes via upregulation in the sex they benefit, downregulation in the sex they harm, or both [35].

### Candidate genes

Our data contain numerous functional genes that are potentially under selection in kiwi. For example, we have identified a kiwi gene that is homologous with chicken *IGF1R* (insulin-like growth factor 1 receptor) and associated with growth and body size in birds [51, 52], as well as seven forkhead box (*FOX*) genes encoding transcription factors critical to proper development and healthy ageing [53–55]. The latter includes *FOXC1* which is associated with ocular development and early onset glaucoma [56–58] and could be related to the unusually high frequency of cataracts in rowi (DKaye, pers comm).

Numerous kiwi genes likely associated with disease resistance were also discovered, including ovotransferrin (*TF*) [59], nine toll-like receptor genes (*TLR*s) [60, 61], and 15 tumor necrosis factor genes (*TNF*s) [62, 63]. *TF* encodes an iron-binding glycoprotein found in avian egg whites, serum, and eggshells that is associated with resistance to a wide variety of infections [64]. Similarly, *TLR* genes function in pathogen recognition and innate immune responses [60]; they are targets of episodic and balancing selection in birds and highly polymorphic [61, 65]. *TNF* genes have roles in controlling inflammation, apoptosis, and cell proliferation and have been associated with numerous and varied diseases in vertebrates [62, 63]. Variation within these genes could be a key component of fitness differences observed among kiwi. The first instances of avian malaria and small pox in kiwi were recently reported and kiwi management provides conditions that could promote the spread of infection via captive rearing of chicks and translocations of adults [66, 67]. *TF* and *TLR*s exhibit antimicrobial properties that could be critical for kiwi survival as well. Kiwi eggs have extremely high levels of antimicrobial proteins relative to other birds, including other ratites [68–70]. They also exhibit high levels of microbial contamination (over 90% of North Island brown kiwi eggs) with bacteria known to cause embryonic death in kiwi and other birds [68, 71]. Thus, microbial infection of eggs and embryos could be a major cause of the hatching failure observed in wild kiwi [21, 72] and potentially have a genetic basis among individual kiwi as well.

Finally, our data also contain genes associated with important avian reproductive and behavioral traits. For example, *GNRH1* (gonadotropin releasing hormone 1) is associated with double yolking [73], *NPY2R* (neuropeptide Y receptor type 2) affects age at first lay and induces precocious puberty in chickens [73, 74], *IGFII* (insulin-like growth factor II) regulates avian ovarian function and egg productivity [75, 76], and *OPN5* (opsin 5) encodes a UV sensitive photoreceptor protein that regulates seasonal reproduction in birds [77, 78]. Variation in these candidate genes may explain the extreme variation observed in reproductive success among the critically endangered rowi or among kiwi species generally. Similarly, exploring patterns of variation at several kiwi genes associated with important avian behaviors may shed light on kiwi ecology and conservation. These include *DRD4* (dopamine receptor D4), which is associated with novelty seeking and exploratory behavior in birds and potentially related to colonization success [79], and *FOXP1* (forkhead box P1), which is hypothesized to be involved in avian song learning and production [80].

## Conclusions

Whole genome studies have been increasingly applied to species conservation since the advent of next generation sequencing [5, 6, 81]. For example, whole genome data have been used to determine that red (*Canis rufus*) and Great Lakes (*C. lycaon*) wolves arose from recent hybridization with other canids due to habitat changes and predator control efforts. This finding will likely shift the focus of conservation management from maintaining the admixed populations to conserving older, evolutionarily independent species [7]. Whole genome studies also recently found significant genetic population structuring and extensive inbreeding within species of great apes (*Pan* spp., *Gorilla* spp., and *Pongo* spp.) as well as pervasive hybridization among individuals in captivity [82]. These finding will provide the basis for genetic management of wild and captive great apes. Studies at the genomic level have also proven powerful for detecting selection and adaptation in endangered species. For example, rapid and positive selection has been shown for traits related to a predatory lifestyle in desert habitats in falcons (*Falco* spp.; beak shape, water conservation, sodium secretion) [8] and to diet shifts and olfaction in endangered giant pandas (*Ursus maritimus*) [9]. Finally, genomics has contributed to conservation via improving the resolution of historical demography studies. For example, Sumatran orangutans (*Pongo abelii*) currently have higher genetic diversity than Bornean orangutans (*P. pygmaeus*) despite a seven-fold lower population size. This pattern is apparently due to an exponential expansion of Sumatran orangutans and a decline in Bornean orangutans after their split ~400 thousand years ago [4].

Our study will allow a similar leap forward in conservation genomics of kiwi by providing a genome-wide resource to investigate their evolution and ecology and inform management. The transcriptome provides a critical complement to understanding an organism’s genome. Thus, we expect the transcriptome assemblies provided here to be valuable in delineating the structure of rowi and LSK genomes once available. Our data contain autosomal and sex linked genetic markers representing both putatively neutral loci and loci under selection. This will vastly improve the current power of population genetics studies of kiwi which to date have been based on 30 microsatellite loci at most (KRamstad, unpublished data) [24, 34, 83]. Our new suite of markers will be used to test parentage, build pedigrees, and resolve relationships among birds relative to their mating and territorial behavior, as well as to improve estimates of inbreeding and the frequency of hybridization among kiwi. They will also improve the power of assignment tests for birds and eggs of unknown provenance, such as unlabeled museum specimens and those confiscated from illegal trade. The sex linked markers we have discovered (particularly those with sex-biased expression) may prove helpful to sex kiwi eggs, chicks, juveniles and remains which cannot be sexed phenotypically. Coupled with nuclear and mtDNA markers, they will also advance our understanding of sex specific dispersal patterns among kiwi, pseudoautosomal inheritance, and lack of dosage compensation in birds [35, 48, 49]. Finally, the numerous functional genes we have identified will allow powerful tests for genotype-phenotype associations in kiwi growth, development, disease resistance, reproduction, and behavior. Functional sequence variation in all of these candidate genes can now be assayed in hundreds of individual kiwi, bringing cutting-edge genomic techniques to bear on conservation of this ancient avian lineage.

## Methods

### Sample collection, preparation, and sequencing

Blood was collected from eight LSK and eight rowi (equal sex ratios). LSK were sampled from Kapiti Island (three males, two females), Long Island (one male, one female) and Zealandia Sanctuary (one female) populations. All LSK were adults when sampled apart from a single juvenile male from Kapiti Island. Adult LSK were sampled on Long Island in Austral autumn (2–4 April 2008) and outside of mating and nesting season in late winter and spring, [38] and all other LSK were sampled in Austral spring (23 Sept-15 Oct 2011) during mating and nesting season. Rowi sampled were part of the Operation Nest Egg program (ONE), which collects eggs from the only remaining wild population of rowi at Okarito Sanctuary, hatches them in captivity, rears them on predator-free Motuara Island until they reach sufficient body size to fend off predators, and then returns them as juveniles to Okarito Sanctuary. We sampled juvenile rowi (4 males, 4 females) inhabiting Motuara Island in Austral spring 2011 (4-6 October) and, thus, prior to breeding age and in the absence of predators or adult rowi. All but one bird was captured in the wild and in healthy condition when sampled. The exception was an emaciated female LSK that was sampled after being found trapped in a ground hole in Zealandia Sanctuary.

Approximately 0.5ml of whole blood was drawn from the metatarsal vein of each bird using a sterile 1ml syringe and 22–26 gauge needle, and added to 125μl K_2_EDTA and 750μl of Trizol-LS. Total RNA was extracted from whole blood using a Trizol-LS extraction (Invitrogen) followed by a HighPure RNA Isolation clean up (Roche). After phase extraction with Trizol and chloroform, an equal volume of 75% ethanol was added to the aqueous phase and the whole mixture applied to a HighPure column; purification of RNA from the column was performed according to the manufacturer’s instructions. Extracts were then treated with RNasin ribonuclease inhibitor (InVitro Technologies) to stabilize the RNA, followed by a DNase treatment to degrade any remaining DNA contamination.

Illumina sequencing was performed by New Zealand Genomics Ltd (Dunedin, New Zealand). RNA integrity was assessed with an Agilent Technologies 2100 Bioanalyzer, which indicated a mean RNA concentration of 64.6ng/ul (range 18–173) and a mean RNA Integrity Number (RIN) of 6.6 (range 6–7.5). Following purification via polyA selection, mRNA libraries were created using the TruSeq™ RNA Sample Preparation Kit (Illumina, San Diego, CA, USA) and pooled in equimolar amounts before loading onto a total of four lanes of an Illumina HiSeq2000 sequencer for 1 × 100bp paired end sequencing to a total depth of 851,254,015 paired reads. Three rowi were initially sequenced to assess the level of hemoglobin transcripts followed by sequencing of the remaining 13 samples.

### De novo assembly and assembly annotation

A small subset of the reads (250,000) were first assembled using the Trinity package [84, 85]. From the resulting contigs, two distinct sequences were identified that were expressed at a high level (~20% of reads) and correlated to hemoglobin subunit genes. The entire dataset was then aligned to these contigs with Bowtie 2.0 [86] and the aligned reads removed. Remaining sequences were assembled *de novo* to create individual and composite transcriptome assemblies for LSK and rowi separately. All transcripts were filtered based on the primary transcript (noted seq1 in Trinity) to remove alternative splicing variants, and searched for an ORF using the in-built Trinity Open Read Frame finding script. Contigs with an ORF of greater than 100 amino acids were mapped against chicken genome protein sequences (Chicken *Ensembl* release78, http://uswest.ensembl.org/Gallus_gallus) and orthologous genes were identified as the BLASTP hit with the highest score and alignment length. Chromosomal location of kiwi genes were predicted to be the same as that of their chicken orthologues based on highly conserved synteny across avian taxa [35, 39, 41]. To verify the integrity of our transcriptome assemblies, we aligned the composite *de novo* LSK assembly at the DNA level with the recently published North Island brown kiwi genome (ENA accession number: GCA 001039765.1) using BLAT [87].

The composite *de novo* LSK assembly was also BLAST searched against the NCBI NR protein database using BLASTx to return the top 20 non-redundant matches per sequence with an e-value cut-off of 1×10^−4^. BLASTx results were then assigned GO labels in Blast2GO Pro (v 2.7.2) [88] with an e-value cut-off of 1e-^6^, an annotation score cut-off of 55, and a GO weight of five. GO annotations were merged with those from an InterPro scan to further improve annotation, and grouped into GO-slim terms to simplify the output. Combined graphs of biological process and molecular function were then produced with nodes containing fewer than 10 sequences filtered out. BLASTx and Blast2GO outputs were uploaded into a MySQL database to facilitate calculation of summary statistics and searches for specific genes.

### SNP identification and validation

All reads for each of the 16 individual birds were mapped as pairs to the LSK composite reference (20,152 genes containing ORF >100 amino acids) using Bowtie 2 [86]. Single nucleotide variants (SNPs) and small insertion/deletions were then identified using GATK version 1.2 [89, 90]. To confidently identify SNPs that distinguish between species or individuals, we filtered the data for a minimum coverage of 50 reads across the variant in each of the birds and, within contigs where there was a density of less than 1 variant per 200bp, selected those markers where at least one individual had a SNP frequency lower than 5% (expected reference genotype) and at least one individual had a SNP frequency greater than 35% (expected heterozygous or homozygous genotype).

### Gene expression analysis

We determined the raw expression of each transcript for each sample by mapping all reads back to the LSK reference assembly and counting the number of reads mapping to each transcript per sample in the Bowtie 2.0 alignment. We normalized the reads by quantile and assessed differential expression with *β*-statistics using functions in the EdgeR package [91]. Expression comparisons were made between species and between sexes. Differences between species were reported where differential expression was ≥ 2 fold and *β*-statistics exceed zero. We did not use a 2 fold threshold in comparing expression differences between sexes, however, as we were specifically testing for 2 fold male-biased expression indicating genes present on the Z chromosome but outside the PAR. Results are given for differential expression between the sexes with and without the 2 fold threshold for comparison where appropriate.

## Additional files

Additional file 1: FASTA file of LSK composite reference transcriptome. Includes 20,152 contigs containing an open reading frame greater than 100 amino acids. (FASTA 45736 kb) Additional file 2: Comma separated file (.csv) of BLAST results and alignment with the North Island brown kiwi genome. Includes the top BLAST hit for each LSK reference contig against NCBI-NR, chicken *Ensembl*, human *Ensembl* database, as well as the contig of the kiwi genome assembly to which each transcript mapped and the percent length mapped. (CSV 6520 kb) Additional file 3: Comma separated file (.csv) of 66,909 SNPs that differentiate between LSK and rowi. Includes SNP position within the reference assembly, reference and alternative alleles, if SNP is untranslated or causes synonymous or non-synonymous substitutions, and predicted chicken chromosome synteny. (CSV 2607 kb) Additional file 4: Comma separated file (.csv) of 12,384 SNPs that differentiate among LSK. Includes SNP position within the reference assembly, reference and alternative alleles, if SNP is untranslated or causes synonymous or non-synonymous substitutions, predicted chicken chromosome synteny, and the SNP frequency in each LSK sequenced. (CSV 921 kb) Additional file 5: Comma separated file (.csv) of 29,313 SNPs that differentiate among rowi. Includes SNP position within the reference assembly, reference and alternative alleles, if SNP is untranslated or causes synonymous or non-synonymous substitutions, predicted chicken chromosome synteny, and the SNP frequency in each rowi sequenced. (CSV 2237 kb) Additional file 6: Comma separated file (.csv) of raw expression levels. The number of reads mapped to each LSK reference contig for all kiwi sampled (CSV 1820 kb) Additional file 7: Comma separated file (.csv) of 3084 transcripts differentially expressed between LSK and rowi. Includes the β-statistic, magnitude of expression difference, and direction of regulation (CSV 100 kb) Additional file 8: Comma separated file (.csv) of 150 transcripts differentially expressed between male and female kiwi. Includes the β-statistic, magnitude of expression difference, direction of regulation (given in males relative to females), and synteny to the chicken genome (where available) (CSV 5 kb)

## Abbreviations

chrZ: chromosome Z
*DRD4*: dopamine receptor D4
*FOX*: forkhead box
*FOXC1*: forkhead box C1
*FOXP1*: forkhead box P1
*GNRH1*: gonadotropin releasing hormone 1
GO: gene ontogeny
*IGF1R*: insulin-like growth factor 1 receptor
*IGFII*: insulin-like growth factor II
IUCN: International Union for Conservation of Nature
LSK: little spotted kiwi
mRNA: messenger RNA
mtDNA: mitochondrial DNA
NCBI NR database: National Center for Biotechnology Information non-redundant database
*NPY2R*: neuropeptide Y receptor type 2
*ONE*: Operation Nest Egg
*OPN5*: opsin 5
ORF: open reading frame
PAR: pseudoautosomal region
SNP: single nucleotide polymorphism
*TF*: ovotransferrin
*TLR*: toll-like receptor
*TNF*: tumor necrosis factor
